# Prevalence, Characteristics, and One-Year Follow-Up of Congenital Cytomegalovirus Infection in Isfahan City, Iran

**DOI:** 10.1155/2016/7812106

**Published:** 2016-12-14

**Authors:** Pegah Karimian, Omid Yaghini, Hossein Nasr Azadani, Majid Mohammadizadeh, Seyed Ali Mohammad Arabzadeh, Atosa Adibi, Hamid Rahimi

**Affiliations:** ^1^Department of Pediatrics, Isfahan University of Medical Sciences, Isfahan, Iran; ^2^Department of Medical Virology, Tehran University of Medical Sciences, Tehran, Iran; ^3^Department of Medical Virology, Kerman University of Medical Sciences, Kerman, Iran; ^4^Department of Radiology, Isfahan University of Medical Sciences, Isfahan, Iran

## Abstract

*Introduction.* Need of neonatal screening for* Cytomegalovirus* (CMV) infection is under debate, in part because of limited data on importance of the disease regarding the prevalence of congenital CMV (cCMV) infection and associated morbidity and mortality. We aimed to evaluate the prevalence and prognosis of cCMV infection in Iran, where there is high maternal seroprevalence of CMV.* Methodology*. This prospective study was conducted in Isfahan city, Iran, from 2014 to 2016. CMV was investigated in urine specimens by using the real-time polymerase chain reaction (RT-PCR) method. CMV-infected infants were examined for clinical and laboratory findings attributed to CMV infection and followed up for one year.* Results.* Among 1617 studied neonates, eight (0.49%) were positive for CMV infection. CMV-infected neonates were more likely to be preterm than noninfected ones (25% versus 4.5%, *p* = 0.0508), and they had lower birth weight. Three out of the eight CMV-infected neonates had transient symptoms at birth. At follow-up, one case had mild hearing loss. Most patients had impaired growth during the one-year follow-up.* Conclusions.* The primary object of this study was determination of prevalence of cCMV infection in Iran as a developing country, which was at the lower range compared with other such countries. cCMV infection may result in short-term impairment in growth.

## 1. Introduction

The contributing factors in transmission of CMV from mother to the fetus have not been well defined [1]; however, the epidemiological characteristics of the population have a rule on it. In a recent meta-analysis of studies of birth or fetal prevalence for CMV infection in different geographic areas, the overall prevalence of cCMV infection was 0.64%, with significant variation worldwide [2]. Based on other studies, cCMV infection prevalence in developing countries also varies from 0.6% to 6.1% [3]. Most of the neonates with cCMV infection are asymptomatic at birth and only 11% have symptoms that can be attributed to CMV infection [4]. Although the mortality rate due to cCMV infection is 5% to 10% in symptomatic infants, 40% to 58% of the cases will develop irreversible complications [5]. Long-term sensorineural deafness is the most common complication of cCMV infection [6]. However, half of all cases are not recognized during neonatal screenings because of the late presentation of this complication up to the age of six-year [4].

Screening strategies help early detection of the CMV-related disabilities such as hearing loss and other neurological complications, which in turn may help to prompt a more efficient treatment and to improve the outcomes [7]. More epidemiological data as well as information on long-term prognosis of symptomatic and asymptomatic cCMV infection is still required to contribute the debates on need of neonatal screening for cCMV infection. The percentage of CMV-infected neonates with symptoms at birth or permanent sequelae may differ between countries or populations with low to moderate positive seroprevalence of CMV in women at reproductive age compared to countries or populations with high positive seroprevalence of CMV [2]. Considering this and scarcity of population-based studies with unbiased sampling and standard diagnostic method in developing countries [3], this study was conducted to determine the prevalence and clinical course of cCMV infection in a representative sample from a large city in central Iran, which has high maternal seroprevalence of CMV [8].

## 2. Materials and Methods

### 2.1. Subjects and Setting

This prospective study was conducted in Isfahan city, Iran, from November 2014 to January 2016. Simple consecutive sampling was done in three provincial healthcare centers (Fadaei, Ebne Sina, and Navabe Safavi) which serve as referral centers for screening of neonatal hypothyroidism in Isfahan. To determine the rate of cCMV infection among all newborns, the sample size was calculated as 1550 cases based on type I error probability of 0.05, estimating prevalence of cCMV as 1% [3], and a precision of 0.5%. All neonates in these centers were included in the study until the intended number of specimens was collected. The study was approved by the Research Ethics Committee of the Isfahan University of Medical Sciences, and the written consent was obtained from all the parents.

### 2.2. Assessments

The necessary data, including maternal and paternal age, education level, and histories of abortion and diseases before and during pregnancy, were collected through interviews. Data on the order of birth, gestational age, gender, weight, height, head circumference, and concomitant diseases of the neonates were collected from their medical records.

#### 2.2.1. CMV Laboratory Assessment

Urine specimens were collected from neonates using urine bags. Specimens were kept in Eppendorf tubes in the refrigerator at 4°C for up to 24 hours and then they were stored in a refrigerator at a temperature of −80°C till the time of real-time polymerase chain reaction (RT-PCR) study. All specimens were tested for CMV using the RT-PCR method in a university-affiliated laboratory with a commercial standard kit (AmpliSens, CMV-screen/monitor-FRT PCR kit, Slovak Republic) in strict accordance with the manufacturer's instructions. Urine specimens were mixed in groups of 20 samples (10 *μ*l from each specimen). In case of having a positive result in each pool, the associated urine specimens were divided into two groups of 10 and PCR test was done on each group separately. This method continued until the single positive CMV sample was detected. The whole testing time was less than 48 hours for each specimen. This approach was applied based on the method used for the detection of blood-borne viruses in transfusion settings [9]. The accuracy of this screening method is equivalent to the standard methods, while it costs less [10, 11].

#### 2.2.2. Clinical Examination

All CMV-positive neonates were evaluated through general physical examination, comprehensive neurological examination, developmental assessment, and ophthalmologic evaluation by fundoscopy all performed by three of authors (Hamid Rahimi, Pegah Karimian, and Omid Yaghini) and an expert ophthalmologist. Laboratory tests, including complete blood count and liver and renal function tests, were done for all CMV-positive cases and hearing evaluation was conducted using auditory brainstem response (ABR) as soon as possible after birth and also at the age of one year in these cases. Cranial ultrasonography was done as soon as possible after birth and computed tomography (CT) scan or magnetic resonance imaging was done in case of having abnormal cranial ultrasonography or having strong evidence of central nervous system involvement [12]. Based on the current recommendations, all positive cases were followed up for one year and visited at ages 2, 4, 6, 9, and 12 months [13]. Birth characteristics and growth parameters (i.e., length, weight, and head circumference) of CMV-infected infants were measured regularly during the study. Considering the neonates gestational age, we used “WHO Anthro for personal computers, version 3.2.2, 2011: Software for assessing growth and development of the world's children. Geneva: WHO, 2010” to determine the *Z*-scores for each of the neonates. Then we plotted all the calculated *Z*-scores on corresponding WHO growth charts as reference standards.

### 2.3. Definitions

In this study, the infants were considered as symptomatic at birth if they had one or more of the symptoms of the CDC National Congenital CMV Disease Registry's case definition [14]. These symptoms must be present at birth or at first clinical examination soon after detection of CMV in urine; however, all of these symptoms are nonspecific and most of them resolved with no specific antiviral therapy. Some of CMV-infected infants had classical CMV symptoms, that is, SNHL or chorioretinitis that persisted during follow-up and considered as sequel of cCMV infection. Delivery at <37 weeks' gestation was considered preterm.

### 2.4. Statistical Analysis

Using the SPSS software for windows version 21.0 (SPSS Inc., Chicago IL., USA), data were analyzed using Fisher's exact test for qualitative variables and Mann–Whitney *U* test for nonparametric data. Descriptive data were presented as mean ± standard deviation (SD), median (range), or frequency (valid percent). A *p* value < 0.05 was considered statistically significant.

## 3. Results and Discussion

### 3.1. Results

A total of 1690 neonates were evaluated during the study period from which 73 were excluded from the study because of missing data, missing specimens, and technical problems during lab analysis. Finally, data of 1617 neonates (56.9% boys) were included in the analysis. All of these neonates had age less than two weeks at the time of detection of CMV in the urine specimens. Seventy-six (4.7%) of the neonates were preterm (with gestational age <37 weeks). Eight neonates (0.49%) whose characteristics are described in Table 1 were CMV-infected. Of these cases, three cases (37.5%) were classified as symptomatic at birth and all of them were admitted in the hospital due to jaundice, suspected sepsis, and respiratory distress syndrome; none of them received specific antiviral treatment for CMV infection and were discharged after improvement of symptoms. However, two of them had neutropenia after discharge from the hospital that resolved up to age 12 months. To minimize radiation exposure, cranial ultrasounds were done for all neonates and it revealed some abnormalities in three CMV-infected neonates, but further brain CT scans and follow-up cranial ultrasounds were normal in these three cases, meaning the first cranial ultrasounds had been done with too much punctiliousness. All the CMV-infected cases passed routine neonatal hearing screening, but mild SNHL with hearing threshold 26–40 decibels was detected in one of them by conducting further ABR testing. One neonate had hyperpigmentation of the retinal pigment epithelium in ophthalmologic examination.

Comparison between CMV-infected and CMV-uninfected infants is shown in Table 2. CMV-infected neonates are more probable to be preterm than uninfected ones (25% versus 4.5%, *p* = 0.0508). Mean birth weight is lower in CMV-infected neonates (mean difference = −295.0 ± 165.5 g, *p* = 0.021).

#### 3.1.1. Follow-Up

Hearing status of the infant with mild sensory neural hearing loss was the same at one year old, and ophthalmologic examination of the infant with hyperpigmentation of the retinal pigment epithelium did not show chorioretinitis or chorioretinal scar on follow-up ophthalmologic exams.

Three infants had neutropenia in initial laboratory work-up; two of them had other symptoms and history of admission in the hospital at first week after birth; in these two cases neutropenia resolved but the last infant with initial neutropenia had persistent neutropenia at age of 12 months, without any history of unusual susceptibility to infectious disease. In cases 1 and 5 spleen was palpable after six months of age and one of them (case 1) had also thrombocytopenia in complete blood count at age 12 months.

**Figure 1:**
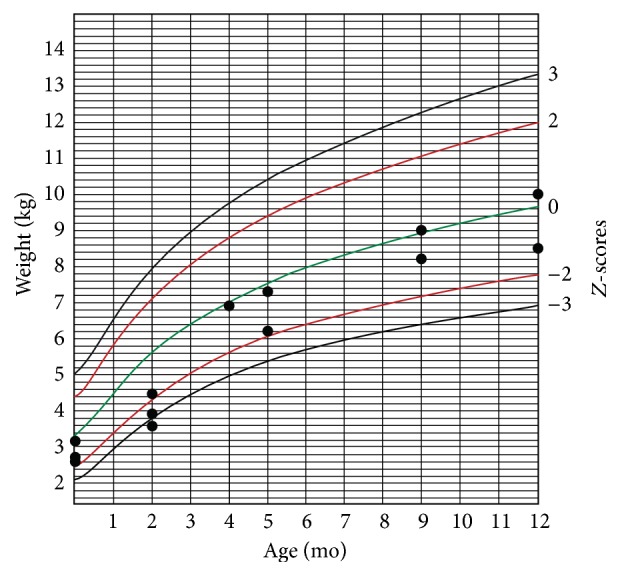
Weight-for-age chart for boys during one-year follow-up.

**Figure 2:**
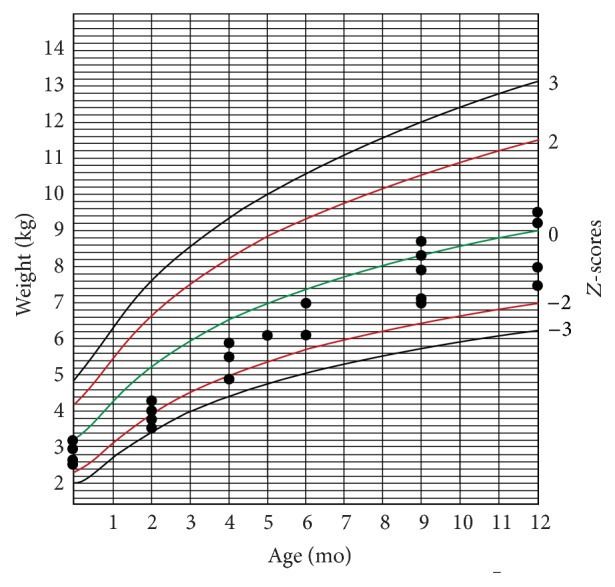
Weight-for-age chart for girls during one-year follow-up.

**Figure 3:**
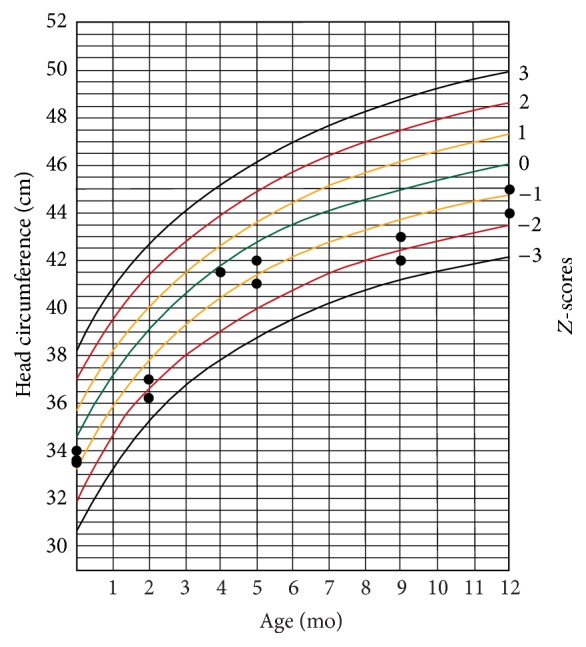
Head circumference-for-age chart for boys during one-year follow-up.

**Figure 4:**
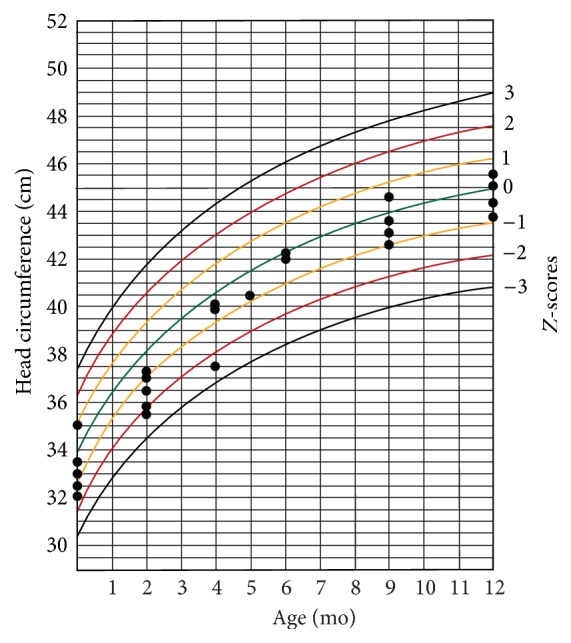
Head circumference-for-age chart for girls during one-year follow-up.

The infants' weight and head circumference were followed for one year after birth (Figures 1–4).

## 4. Discussion

There is little information about the prevalence and natural history of cCMV infection in developing countries, using urine RT-PCR method in this population-based study with a fairly large sample size; we found a cCMV infection rate of about 0.5%, which is lower than the reported range of other developing countries [3]. In previous limited studies, the prevalence of cCMV infection in Iran has been reported from 0.3%, examining saliva specimens [15] to 4% with PCR study of umbilical cord blood [16]. Anti-CMV IgG seroprevalence in pregnant women in Iran has been reported between 70% and 98% while anti-CMV IgM antibodies have been in serum samples of 2.5% to 4.3% of these mothers [17]. It means that most of the women in our country have been infected with CMV before pregnancy. Although some investigators believe that congenital CMV infection in developing countries is most likely caused by nonprimary maternal infections [18], maternal seroprevalence accounts for less than one-third of the difference in prevalence of cCMV and the epidemiology of cCMV does not follow a clear pattern in all parts of the world. In addition, a large variation can be observed within each country in the birth prevalence of CMV [2].

Infants with cCMV infection are mostly asymptomatic at birth and only about 11% have symptoms that can be attributed to the infection [2]. In neonates whose infection is due to secondary maternal infection (the most probable scenario in our country) symptoms are even less likely to be present at birth (less than 2%) [19]; however, 37.5% of cCMV-infected neonates in our study were considered symptomatic at birth, and all of them were admitted in the hospital in the first week of life and improved without receiving anti-CMV therapy. Despite the fact that many of the signs, symptoms, or laboratory abnormalities proposed for diagnosis and classification of cCMV in the literature [14] and in this study are nonspecific, lack of any other explanation for these problems in these three cCMV-infected neonates in the limited work-up done made us consider these symptoms to be probably due to cCMV infection. Similar to a meta-analysis on epidemiologic results of systematic CMV screening on fetuses or live newborns [2], we found a possible association between preterm delivery and cCMV infection, as well as lower birth weight in infected neonates compared to uninfected ones; though some other studies found no evidence of an excess of preterm births in cCMV-infected neonates [20–23]. However, these findings are not pathognomonic for cCMV infection and according to available guidelines, no antiviral therapy was needed in such cases [12, 24, 25].

Congenital CMV infection is a major cause of childhood hearing loss and other neurodevelopmental disabilities such as cognitive and visual impairments [7]. Mild sensory neural hearing loss was present and persistent in one out of the eight (12.5%) infected neonates in our study, which is in the range reported by other studies in asymptomatic infants [6]. Case series of infants with asymptomatic cCMV infection and hearing loss treated with ganciclovir and valganciclovir have reported improvement in hearing loss with treatment [26, 27]. So antiviral therapy was recommended for this infant, but her family did not accept the treatment, and hearing status of the infant was the same at one year old.

Another finding in our study was that most of the infected neonates had abnormal growth during one-year follow-up. Considering the fact that, after the infancy period, environmental factors have greater influence on physical growth of children, we followed up cCMV-infected children up to age 12 months. Several investigators reported no statistic difference in physical growth of children with cCMV infection and cCMV-uninfected children [28–30]. Ivarsson et al. followed a group of children with cCMV for about 10 years and found that those with primary infection were marginally shorter than standard reference at 1 year and 2 years old. However, there was no strong evidence of any relationship between the infection and short stature [31]. Borderline microcephaly in symptomatic CMV-infected infants is associated with adverse long-term neurodevelopmental and cognitive outcomes in these infants [32, 33], and it may therefore indicate the necessity of screening and preventive strategies.

Although cCMV infection is rare, the incidence is not less than some other conditions, such as congenital hypothyroidism with an incidence of 1 : 748 births in Iran, which is currently included in the national screening programs at birth [34]. It is a leading cause of SNHL, neurodevelopmental delay, and vision impairment [7] and disability associated with cCMV infection is as common as some other well-known conditions such as Down syndrome [35], and its economic burden is considerable [7]. Although most of the neonates with cCMV infection were found asymptomatic in our study, these infants are still at risk of long-term complications such as sensorineural hearing loss and neurodevelopmental delay [36]. One of the strategies to reduce this burden is to use a suitable screening method such as pooled urine specimens' examination's technique, as we used in our study, because it is noninvasive, sensitive, and specific and has low cost for universal neonatal screening for cCMV infection [7]. In a recent report from Japan, 12 symptomatic cCMV cases (out of 6348 screened newborns) received antiviral treatment from which five cases (42%) had normal development after at least one year of follow-up [37]. Accordingly, early diagnosis and treatment of cCMV may improve the outcomes in symptomatic infants. In contrast to developed countries whose considerable epidemiologic data are available [7], the natural history and late complications of cCMV in developing countries such as our country are still unknown. However, the beneficial impacts of universal neonatal screening for cCMV in developed countries cannot be generalized to other nations [7]. We can come to a clear conclusion only after more epidemiological and particularly prospective studies of cCMV are done in our country.

Our study had some limits. Although our studied group was a good representative of cCMV infection's epidemiology in neonates in a large city of Iran, the number of positive cases was not enough for the evaluation of risk factors associated with cCMV and larger studies with longer follow-up are required so that we can compare outcomes between symptomatic and asymptomatic infants and the natural history of cCMV infection in a developing country. In addition, we did not determine the status of maternal seroprevalence of the affected infants; whether such data could provide better evidence on transmission risk of primary and secondary maternal infections and would have prognostic value is not clear.

## 5. Conclusions

Using pooled urine specimens' examination technique, we found a prevalence of 0.5% for cCMV infection in Isfahan, Iran, which is in the lower range of its prevalence in other developing countries. In this study 37.5% of cCMV-infected neonates had transient symptoms at birth. One-year follow-up of the affected neonates showed impaired growth in most cases; their head circumference notably remained borderline lower than reference standard at this age. Sensory neural hearing loss was present in one out of the eight patients. Further studies with larger sample size and longer follow-up duration are still required before coming to a clear conclusion on the necessity of universal screening of the newborns for cCMV infection in our country and for treating of the affected patients.

## Figures and Tables

**Table 1:** Maternal and neonatal characteristics in infant with congenital *Cytomegalovirus* infection.

Case number	1	2	3	4	5	6	7	8
Gender	Female	Female	Male	Female	Female	Male	Male	Female
Gestation age (week)	39	39	38	39	38	35	35	38
Birth weight (g)	2950	2610	3140	2520	2650	2600	2700	3180
Birth weight for age (*Z*-score)	−0.63	−1.45	−0.43	−1.68	−1.35	0.25	0.49	−0.11
Birth height (cm)	48	49	50	45	46	49	49	48.0
Birth head circumference (cm)	35	32	34	33.5	33	33.5	33.6	32.5
Birth head circumference for age (*Z*-score)	0.95	−1.59	−0.36	−0.32	−0.74	1.06	1.12	−1.59
Reason for hospitalization at first week of life	—	—	—	Jaundice at birth, normal LFT	—	Jaundice, RDS	Poor feeding	—
Maternal comorbidities	None	None	None	None	None	GDM, anemia	GDM, anemia	None
Urine CMV DNA (copies/ml)	146	149775	1057	239541	535	39850	3565302	7095
Abnormal physical examination (from 6 months afterward)	Palpable spleen	—	—	—	Palpable spleen	—	—	—
Abnormal laboratory results (initial)	—	—	Neutropenia^a^	Neutropenia	—	Neutropenia	—	—
Abnormal laboratory results (at 1 year of age)	Thrombocytopenia^b^	—	Neutropenia	—	—	—	—	—
Cranial ultrasound	Subependymal fine calcification	Normal	Ventricular calcification	Normal	Focal subependymal calcification	Normal	Normal	Normal
Brain CT scan	Normal	—	Normal	—	Normal	—	—	—
Audiometry (initial and at 1 year of age)	Normal	Normal	Normal	Normal	Normal	Normal	Normal	Mild SNHL^c^
Ophthalmologic examination	Hyperpigmentation of retinal pigment epithelium	Normal	Normal	Normal	Normal	Normal	Normal	Normal
Antiviral therapy	—	—	—	—	—	—	—	—

CMV: *Cytomegalovirus*; GDM: gestational diabetes mellitus; LFT: liver function test; RDS: respiratory distress syndrome; SNHL: sensory neural hearing loss.

a: defined as absolute neutrophil count 500–1000/mm^3^; b: defined as platelet count less than 150000/mm^3^; c: defined as hearing threshold 26–40 dBHL.

**Table 2:** Comparison of maternal and neonatal characteristics between *Cytomegalovirus*-infected and uninfected infants.

Characteristics	CMV-infected neonates (*n* = 8)	Uninfected neonates (*n* = 1617)	*p*
Sex, number (%)			
Male	3 (37.5)	920 (56.9)	0.301
Female	5 (62.5)	697 (43.1)	
Rout of delivery, number (%)			
Vaginal delivery	3 (37.5)	597 (36.9)	>0.999
Cesarean section	5 (62.5)	1020 (63.1)	
Gestational age, (week) number (%)			
<37	2 (25.0)	73 (4.5)	0.0508
≥37	6 (75.0)	1544 (95.5)	
Birth weight (g) (mean ± SD)	2793.7 ± 258.8	3088.8 ± 467.8	0.021
Birth height (cm) (mean ± SD)	48.0 ± 1.6	49.2 ± 2.6	0.064
Birth head circumference (cm) (mean ± SD)	33.4 ± 0.9	34.4 ± 1.8	0.039
Mother age (years) (mean ± SD)	26.8 ± 5.6	28.9 ± 4.7	0.210
Number of delivery (median [range])	1 [1-2]	1 [1–9]	0.099
Family members (median [range])	3 [3-4]	3 [1–9]	0.102
